# The Stigma Surrounding Opioid Use as a Barrier to Cancer-Pain Management: An Overview of Experiences with Fear, Shame, and Poorly Controlled Pain in the Context of Advanced Cancer

**DOI:** 10.3390/curroncol30060437

**Published:** 2023-06-17

**Authors:** Hannah Harsanyi, Colleen Cuthbert, Fiona Schulte

**Affiliations:** 1 Department of Community Health Sciences, University of Calgary, Calgary, AB T2N 1N4, Canada; 2 Faculty of Nursing, University of Calgary, Calgary, AB T2N 1N4, Canada; 3 Division of Psychosocial Oncology, Department of Oncology, University of Calgary, Calgary, AB T2N 1N4, Canada

**Keywords:** cancer pain, opioid use, advanced cancer, symptom management, stigma

## Abstract

Cancer-related pain affects a majority of patients with advanced cancer and is often undertreated. The treatment of this pain is largely reliant on the use of opioids, which are essential medicines for symptom management and the maintenance of quality of life (QoL) for patients with advanced cancer. While there are cancer-specific guidelines for the treatment of pain, widespread publication and policy changes in response to the opioid epidemic have drastically impacted perceptions of opioid use. This overview therefore aims to investigate how manifestations of opioid stigma impact pain management in cancer settings, with an emphasis on the experiences of patients with advanced cancer. Opioid use has been widely stigmatized in multiple domains, including public, healthcare, and patient populations. Physician hesitancy in prescribing and pharmacist vigilance in dispensing were identified as barriers to optimal pain management, and may contribute to stigma in the context of advanced cancer. Evidence in the literature suggests that opioid stigma may result in patient deviations from prescription instructions, which generally leads to pain undertreatment. Patients reflected on experiencing shame and fear surrounding their prescription opioid use and feeling uncomfortable communicating with their healthcare providers on these topics. Our findings indicate that future work is required to educate patients and providers in order to de-stigmatize opioid use. Through alleviating stigma, patients may be better able to make decisions regarding their pain management which lead to freedom from cancer-related pain and improved QoL.

## 1. Introduction

Pain is defined by the International Association for the Study of Pain (IASP) as an “unpleasant sensory and emotional experience associated with, or resembling that associated with, actual or potential tissue damage” [1], demonstrating that pain is a complex biopsychosocial symptom. Cancer-related pain (CRP) affects more than 65% of patients with advanced cancer [2] and is one of the most common [3] and highest priority symptoms for these patients [4,5]. CRP impacts multiple aspects of wellbeing, including physical, emotional, and interpersonal domains, and improper management can thereby have negative impacts, such as sleep disturbance, depression, distress, and diminished quality of life (QoL) [6]. Manifestations of pain are not only physical, but are inextricably linked to patient perceptions, which are modulated by mood, culture, psychological wellbeing, and personal beliefs [7,8]. Due to the complex nature of CRP, the provision of appropriate pain management is a difficult task, but is essential for maintaining QoL for a majority of patients with advanced cancer [9].

The population of patients with advanced cancer is largely understudied [7], and these patients have unique needs as a result of facing incurable prognoses and living with cancer as a chronic disease. Over recent years, more advanced treatment options have allowed patients with terminal diagnoses to have significantly increased survival times [7]. For example, among young women with metastatic breast cancer, 5-year survival rates increased from 45.6% to 56.5% from 2005–2009 to 2010–2015, while survival rates remained relatively stable among women with non-metastatic disease over the same period [10]. In many cases, patients with advanced stage disease are now living with cancer and its effects for many years [11] and may experience prolonged existential burden, psychosocial distress, and physical symptoms as a result. Patients with advanced cancer commonly report high levels of depression, anxiety, and hopelessness [12], in addition to physical symptoms including pain, nausea, fatigue, and dyspnea [13].

The goal of treatment for these patients is generally palliative, focused on the alleviation of symptoms, rather than curative-intent. In these cases, the healthcare system’s primary role is centered on providing symptom control and maintaining patient QoL throughout the disease trajectory. Due to a high prevalence of pain, coupled with increasing survival times and differing treatment priorities, barriers to effective symptom management are particularly impactful for patients with advanced cancer [14]. This paper therefore aims to provide an overview of the crucial role of opioids for pain management in the context of advanced cancer, and explore how perceptions of opioid use in cancer settings have been influenced by extensive publication of the opioid crisis. Based on these findings, the review will provide considerations for clinical practice and future directions.

## 2. Methods

A narrative review was completed using keyword searches on PubMed, EMBASE, and Google Scholar. Keywords included ‘cancer’, ‘malignant’, ‘metastatic’, ‘advanced’, ‘opioid’, ‘pain’, ‘cancer-related pain’, ‘pain management’, ‘stigma’, and ‘opiophobia’. Included articles focused on perceptions of opioid use for the treatment of pain among patients with advanced cancer. Abstract-only and non-English articles were excluded.

## 3. The Role of Opioids in Cancer Pain

Opioids are considered to be a gold-standard therapy for cancer pain management, and the World Health Organization (WHO) recommends them as the first choice for the treatment of moderate-to-severe CRP [15]. Opioids act as analgesics by binding receptors on the nociceptive pathway and thereby reduce perception of pain at the somatosensory cortex [16]. Common opioid therapies used in the treatment of CRP include morphine, hydromorphone, oxycodone, codeine, and fentanyl. Opioid analgesics have been shown to be highly effective [17,18], and are designated as essential medicines for palliative care [19]. Clinically meaningful alleviation of CRP can be achieved through opioid use in approximately 80% cases [20], and while multimodal management strategies are needed for the remaining approximately 20% of cases [21], opioid therapies are an indispensable resource for pain and symptom management among patients with cancer. Guidelines for the treatment of cancer-related pain recommend that opioids be prescribed to patients with moderate-to-severe pain, unless contraindicated, and should be prescribed at the lowest dose necessary to achieve adequate analgesia [22]. Additionally, clinical guidelines recommend that all patients with advanced cancer receive access to specialty palliative care services which has increased expertise and focus on pain and symptom management [23]. Despite these guidelines outlining the appropriate treatment of CRP, meta-analyses have demonstrated that more than 30% of patients with cancer are undertreated based on their pain severity [24,25]. While pain undertreatment appears to be less prevalent among patients with advanced disease, in studies including a majority of metastatic patients the rate of undertreatment remains high, affecting approximately 20% of patients [24].

## 4. The Opioid Epidemic and Changes in Opioid Prescribing 

North America is facing an ongoing opioid epidemic which poses one of the most challenging and pressing public health issues of our time [26]. The Government of Canada has reported more than 30,000 opioid toxicity deaths from 2016 to 2021, and a worsening of the overdose crisis over time [27]. In recent years, numerous updated guidelines have been published with the intent of curbing the prescribing of opioids in response to high levels of non-medical opioid use (NMOU) and associated adverse effects [28,29]. However, it is important to note that these guidelines were not intended to impact patients experiencing CRP and were specifically established for the treatment of non-cancer pain [30].

Despite the intended limited scope of updated guidelines, declines in opioid prescribing have been observed among oncologists in recent years [31,32,33,34], and these declines have been particularly pronounced among patients with metastatic cancers. From 2011 to 2017, opioid prescriptions for patients with bone metastasis were found to decline significantly, in terms of both proportion of patients receiving prescriptions as well as dosage level [33]. It is difficult to determine if these changes represent an appropriate correction in over-prescribing by certain practitioners, greater reliance on alternative methods of pain management, or could represent a concerning exacerbation of issues of pain undertreatment [35]. What is clear is that the ongoing opioid epidemic has led to increased stigmatization regarding the use of opioids, with some research proposing two concurrent opioid epidemics: one resulting from illicit opioid use and overdose deaths, and another resulting from ‘opiophobia’, characterized by reduced access to opioids and fears relating to their use [36].

## 5. What Are the Risks of Opioid Use among Advanced Cancer Patients?

There is a variety of evidence indicating that there are significant differences between cancer and non-cancer populations in terms of the risks of opioid use. The lifetime prevalence of opioid-use disorder among non-cancer patients prescribed long-term opioid therapy has been estimated at 41.3% [37], compared to an estimated prevalence of 8% among patients with cancer-related chronic pain [38]. In terms of overdose deaths, there were 8.97 opioid deaths per 100,000 people among the general population in 2016, compared to 0.66 opioid deaths per 100,000 among cancer patients [39]. Given the differences between these populations, it is clear that prescribing practices or guidelines should not be extrapolated from the general population to patients with cancer.

While there are differences between cancer and non-cancer populations, consideration of the risks related to opioid use is still relevant when prescribing opioids to cancer populations. The older literature suggests that NMOU among patients with cancer is low compared to the general population [40,41]. However, while commonly cited, these studies are outdated and may no longer be relevant in the opioid epidemic era. A more recent review indicated that approximately 20% of patients with cancer are at high risk for opioid use disorder [42]. Similarly, a recent study of 1554 patients taking opioids for cancer pain found that 19% developed NMOU behaviors [43]. When specifically investigating patients with advanced cancers, a study found that 18% were diagnosed with chemical coping [44]. While there are a variety of metrics for evaluating NMOU which are difficult to compare, it is increasingly evident these issues are applicable to the cancer community, including those with advanced cancer.

Chronic opioid use, which may be a risk factor for NMOU [45], has been defined as use of medically prescribed opioids for at least 90 days [46]; this outcome has not commonly been considered a concern for patients with advanced cancer given their historically short survival times. The significant increase in survival times among this population means that consideration of chronic use has become applicable, and studies on this topic are timely, if not overdue [35,47]. Among opioid-naïve patients after curative-intent surgery for cancer, the risk of new persistent opioid use has been estimated at approximately 10.4% [48]. There are no population-based estimates for the risk of chronic opioid use in patients with advanced cancer. The inability to provide patients with this information may lead to exaggerated fears regarding long-term opioid use, and the exclusion of these patients from studies may be damaging, rather than shielding, in terms of patient perceptions.

Further concerns for patients receiving opioid therapy include adverse effects such as the development of tolerance, physical dependence, cognitive dysfunction, constipation, and nausea [49]. While there are guidelines relating to the management of opioid-induced adverse effects among patients with cancer [50,51,52], there is limited information on the incidence or prevalence of effects related to dependence and tolerance. The onset of adverse effects, as well as their potential for alleviation, are important considerations for patients, who need to weigh the tradeoffs between these symptoms, their impact on wellbeing and functioning, and the potential for experiencing freedom from pain.

In many cases, opioid dose escalations are required to maintain analgesic efficacy, which may be indicative of opioid tolerance or the pain hypersensitivity characteristic of opioid-induced hyperalgesia (OIH) [53,54]. In cancer populations, the requirement for dose escalation is commonly attributed to disease progression, rather than increased tolerance or OIH [55,56]. While many patients may experience increasing levels of pain as a result of disease progression, more research is needed to better understand the contribution of opioid tolerance and OIH in dose escalation and prolonged opioid use for patients with advanced cancer [56].

An additional consideration for opioid prescribing to patients with cancer involves the immunomodulatory effects of opioids and their potential impact on disease progression or metastasis. The literature on the role of opioids in cancer remains contested, largely due to the complexity of the direct and indirect interaction of opioids with immune functioning, inflammation, and metastasis [57]. Long-term use and higher opioid dosages can reduce immune functioning and may impact its cancer-suppressing activity [58]. These risks require further research and review to elucidate the role of opioids in cancer promotion and suppression, as well as the clinical relevance of these effects [59].

A lack of well-validated and specific information on the risks related to opioid use among patients with advanced cancer leaves patients and providers to guess and extrapolate regarding the trade-off between benefits and risks of opioid use. More research is required to understand these risks for patients with advanced cancer, as well as how they differ from broader cancer and non-cancer populations. This information could serve to alleviate uncertainty, which breeds fear and may be a contributor to stigmatization of opioids in this setting. The limited research on these outcomes means that implementation of a benefit-to-harm framework [60,61] may not be sufficiently informed for this population, and more work is needed to effectively implement risk assessment for patients with advanced cancer.

In recent years oncology-specific guidelines have encouraged careful risk assessment when prescribing opioids to patients with advanced cancer [60,62,63,64]. While the consideration of the risks of opioid use is important, assessment without interference from socioeconomic or racial biases poses a significant challenge to clinical oncologists. It has been demonstrated that physicians’ implicit biases may lead to disproportionate pain undertreatment in vulnerable groups [65,66,67]. Risk assessment must therefore be carefully considered, and further developments which acknowledge the intrinsic biases with which providers approach shared decision-making opportunities are needed to improve these guidelines.

Risk assessment in this setting must additionally be considered with reference to issues of pain undertreatment. Under risk assessment recommendations, oncologists’ assessment of risk leads to a triage by risk process, wherein the clinician may opt to prescribe opioids, not prescribe opioids, or initiate opioids with risk-mitigation strategies and monitoring of drug-related behaviors [63]. Hesitancies in opioid prescribing may result from risk assessment which is not properly informed by a benefit-to-harm framework and may be influenced by stigma. A strong emphasis on risk assessment or mitigation, particularly without well-validated information among patients with advanced cancer, may act to further stigmatize opioid use, and act as a barrier to pain management in a population which is already subject to pain undertreatment.

## 6. Defining Opioid Stigma

Stigma has been extensively studied and theorized following the publication of Goffman’s seminal text [68], which defined stigma as “possessing and attribute which makes (someone) different from others” and “of a less desirable kind”. Since then, a variety of academic and colloquial meanings have been attached to the concept. For the remainder of this review, we will refer to stigma as existing when (1) “people distinguish and label human differences”, (2) “dominant cultural beliefs link labeled persons to undesirable characteristics”, and (3) “labelled persons are placed in distinct categories so as to accomplish some degree of separation of ‘us’ from ‘them’ [69]. Extensive literature has demonstrated that stigma follows social structures including race, socioeconomic status, and gender, and can contribute to inequities in health [70]. In healthcare settings, stigmatization can negatively impact patients’ self-perception, social support, and willingness to seek services, and these effects may be heightened by further stigmatizing behavior of healthcare professionals [71]. Stigma may be categorized as either public stigma or self-stigma, and it has been shown the development of self-stigma follows the establishment of public stigma [72,73].

In relation to opioids, it has been argued that the stigmatized history of opioids has defined the current epidemic, rather than arising as a parallel process in response to the crisis [74]. Regardless of the nature of the causal relationship between the modern opioid crisis and opioid stigma, it is clear that both medical and non-medical opioid use are affected by dimensions of stigma in public, clinical, and internalized domains [75]. Opioid stigma may be experienced from external sources, including media, peers, or healthcare providers, as well as internally, relating to patients’ own feelings of fear or shame.

## 7. Public Stigma and Opioid Use

Over the past 20 years, news media coverage on the opioid epidemic and prescription opioids has dramatically increased, with more than 35,000 online news reports from 2018 to 2019 in the United States alone [76]. News stories discussing the opioid epidemic have been found to be far more likely to include stigmatizing terms, such as addict, compared to less stigmatizing alternatives, such as substance use disorder [77]. Public attitudes towards individuals with opioid use disorders have been found to have high levels of stigma, even among individuals with personal experiences relevant to opioid use [78]. The highly publicized and stigmatized presentation of the opioid crisis has resulted in high levels of stigma within the public consciousness, and these perceptions have permeated the oncology setting [75], creating an environment which encourages of the formation of self-stigma among patients with cancer.

## 8. Discussion: Opioid Stigma in Cancer Settings

### 8.1. Pharmacovigilance & Indications of Opioid Stigma among Healthcare Professionals

The WHO identifies pharmacovigilance as aiming “to enhance patient care and patient safety in relation to the use of medicines, and to support public health programs by providing reliable, balanced information for the effective assessment of the risk-benefit profile of medicines” [79]. This concept is essential for safe and effective prescribing practices, including the prescribing of opioids for cancer pain management. However, disproportionate focus on the risks related to certain pharmaceuticals can lead to misuse of a benefit-to-harm framework, and thereby cause reluctance to enact optimal prescribing practices. The wide-reaching nature of the opioid epidemic may mean that both prescribers and patients are more likely to have personal experiences with opioid misuse or addiction, in addition to extensive press coverage, which may impact ideas about the utility of and risks associated with opioid use. These perceptions may not be consciously recognized by physicians, and may lead to unintended consequences such as limited access to opioids, even among patients with advanced disease who are recognized to be in great need of these medicines.

Healthcare professionals often have very differing views on the role of opioids in cancer pain management, which may be related to their discipline and the type of care they provide [80]. For example, palliative care physicians may have very different perceptions of the dosage level and duration of prescription that is appropriate for patients, when compared to surgeons who typically prescribe opioids for acute post-operative pain [80,81].

There are several studies which evaluate the diverse physician perceptions of opioid use in the context of cancer pain management. In a survey of more than 600 U.S. medical oncologists, a majority of respondents described themselves as less conservative in prescribing opioids than their peers and reported physician reluctance in opioid prescribing as a barrier to optimal pain management [82]. Despite reports indicating relatively high confidence among oncologists, it has been shown that physicians most satisfied with their abilities to manage CRP were actually the most reluctant in their use of strong opioids [83]. In another study, 41% of radiotherapists reported staff reluctance to prescribe opioids as a barrier to pain management [84]. Nearly a third of Eastern Cooperative Oncology Group physicians endorsed waiting until patient prognosis was 6 months or less before starting maximal analgesia [85], and a survey of general health care providers found that more than 70% of respondents had concerns about NMOU in patients with cancer [86]. In a review of physician-related barriers to cancer pain management, findings indicated that physician reports consistently included concerns about high doses and side effects of opioids [87]. Each of these attitudes may represent a manifestation of opioid stigma and may act as contributor to pain undertreatment by adversely impacting pain management outcomes for patients with cancer. These studies demonstrate that it is common for healthcare professionals to endorse their own behaviors regarding cancer pain management, but reports are commonly critical towards peers, and hesitancy in opioid prescribing is commonly identified as a barrier to optimal pain management. While acknowledgement of the risks of opioid use is important for the essential practice of pharmacovigilance, in light of persistent issues of pain undertreatment in cancer populations, the question needs to be raised as to whether this vigilance has become over-rigorous due to increased stigmatization of opioid use.

While the adverse effects of the opioid epidemic are most prevalent in North America [88], manifestations of opioid stigma have been observed globally. Among physicians managing cancer pain across 10 Asian countries, excessive regulation and patient fears of addiction were identified as key barriers to opioid prescribing and pain management [89]. In a survey of physicians in Cyprus, 70% of respondents identified opiophobia as a barrier to the appropriate management of cancer-related pain [90]. Strict regulation on opioid use was identified as a barrier in Palestine and Qatar [91,92], and, similarly, across Europe excessive regulatory barriers in the accessibility of opioids have been identified [93], which may negatively impact the management of cancer pain.

Dimensions of opioid stigma expressed by healthcare professionals are further affected by patient demographics, which may result in differential pain management that is prejudiced against minority or disadvantaged groups. Studies suggest that healthcare providers’ distinction between patients who have legitimate or illegitimate pain tends to be influenced by class and racial characteristics [94,95], similar to the delineation of stigma within these groups. Qualitative accounts have also demonstrated that healthcare providers are perceived to have personal biases related to the risks and benefits of long-term opioid therapy that are shaped by personal experiences and patient characteristics, including race and housing status [80]. Race and insurance type have both been found to be independently associated with the type of opioid prescribed to cancer outpatients [96], and there is strong evidence of racial disparities in pain burden and management in cancer settings [97]. Stigma is inextricably related to these health disparities, as minorities and economically disadvantaged groups are easily stereotyped and assigned undesirable characteristics that are distanced from the “us” group. For example, Indigenous peoples may experience elevated external stigma due to an increased prevalence of substance use disorders, and there is significant discrimination against Indigenous peoples in healthcare settings [98]. This stigma has been shown to influence healthcare delivery and may lead to disparities in opioid prescribing and adequate pain treatment [99].

In addition to evidence related to physician accounts, patients with advanced cancer report that their opioid use is stigmatized by healthcare providers, particularly those outside the oncology setting [100]. Patients with cancer commonly experience difficulties in filling opioid prescriptions caused by the pharmacy or pharmacist [84], and report not only increased regulatory obstacles for filling opioid prescriptions, but also increased scrutiny from pharmacy staff that is perceived as judgmental and humiliating [101,102]. Oncologists have described logistical issues with prescribing opioids, including increased oversight and decreased comfort in prescribing, due to restrictive non-cancer regulations that impact their practice [103]. In contrast to issues accessing opioids, patients have reported deferring pain management decisions to oncology providers who have advised them to take opioids [63]. Similarly, patients have reported feeling that oncology clinicians are quick to prescribe opioids without providing sufficient information [104]. These barriers in patient-provider communication and education can lead to patients feeling that they have to self-manage their pain, and this self-management is commonly guided by personal stigma-related perceptions surrounding opioids.

### 8.2. Self-Stigma: Patients’ Experience with Internalized Stigma and Opioid Use

Poor opioid adherence has been reported among patients with cancer, and research has found consistent underutilization of opioids by patients with cancer pain [105,106]. A study comparing longitudinal barriers to cancer pain management found that 40% of patients reported inadequate use of analgesics, and this high rate of inadequate management has been a consistent issue for the past 20 years [107]. This study additionally found that patient concerns about the risks of opioids have increased over this period. In a study of Taiwanese patients with cancer, negative beliefs regarding opioids were found to be significantly associated with poor analgesic adherence [108]. Stigma is one of the most significant patient-identified barriers to adequately managing pain, manifesting as internalized feelings of fear, shame, and discrimination based on their use of opioid analgesics [100,107]. These patient beliefs have been identified as a significant factor in choices related to analgesic use, which are generally modified toward lower dosage levels that fail to control pain [100,102,109].

Fear is one of the most commonly reported feelings relating to opioid use among patients with advanced cancer, and may arise from concerns regarding addiction, dependence, diminishing opioid efficacy, and side effects [100,102,104,109,110,111,112]. Concerns of addiction were found to be among the greatest patient-perceived barriers to cancer pain management in a review of both Western and Asian cancer patients [113]. The opioid epidemic has been identified as foundational to patients’ beliefs regarding the use of opioids, and media or news coverage are contributors to patient fears [102]. Patients commonly view opioid use as an ethical issue and see taking pain medication as “caving in” [102]. Patients may attempt to gain control over their addiction fears through opioid-restricting behaviors, including dose reductions or avoidance of long-acting or strong opioids [102,104,110]. Another recurring contributor to fear includes the belief that proactive or earlier use of opioids would result in inability to achieve relief from pain “when they really need it” [110], demonstrating worries relating to the development tolerance. Patients also expressed that they avoided taking opioids for fear that dependency would develop, and that opioid use would become daily or routine [110,114]. Strong opioids were largely viewed as a “last resort”, and patients reported opioid-avoidant behaviors until pain became totally unbearable or required hospitalization [102,112].

Beyond fears regarding the risk of opioid use, patients often feel shame or guilt, which are constructs of the stigma surrounding opioid use. While it may be viewed as rational that patients have concerns about opioid dependency or side effects, feelings of shame and guilt, especially among patients who are following medical instruction and gaining clinical benefit from opioids, are largely detrimental. Even when partaking in opioid-restricting behaviors, patients express feeling morally compromised and experiencing guilt relating to substance use [102]. It was common for patients to draw false equivalencies to other behaviors, such as smoking, drinking, or other drugs [102,111], and these comparisons contribute to increasing feelings of shame. Patients commonly used highly sensationalized terms, such as addict and junkie [100,102,110], which reflect their internalized stigma against individuals with opioid use disorders. The public perception of opioids as “bad”, and the idea that people who take opioids are also “bad”, is pervasive among patients with advanced cancer and extends to their ability to perceive themselves as good people who deserve relief from pain [100,102]. The shame experienced by these patients is a direct result of opioid-related stigma and impacts not only patients’ ability to manage their pain, but also their psychological wellbeing and self-regard.

A final major theme identified from studies investigating self-stigma among patients with advanced cancer is discomfort with sharing concerns with providers, and feelings that they will be perceived as a “pill-seeker” [102]. Patients reported avoiding conversations with their healthcare providers, for fear of being judged or undergoing scrutiny regarding the legitimacy of their pain and need for opioids [102]. In the alternate case, patients displayed avoidant behaviors to healthcare professionals who were encouraging opioid use, feeling uncomfortable with sharing concerns about addiction and/or related noncompliance with prescription instructions [104,114]. In either case, the effective communication between patient and provider was compromised, and ability to engage in productive conversations, including implementing a benefit-to-harm framework for shared-decision making, were inhibited.

### 8.3. Future Directions

Attempts to destigmatize patient perceptions of opioids can remove a barrier from the effective management of pain and allow patients to feel more comfortable with adequately managing their pain. While there is a growing interest in investigating the manifestations of opioid stigma in this population, there is a general lack of evidence on how these issues may be combatted. Additionally, the majority of research on patient experiences of opioid stigma has been conducted in the United States, and more research would be useful to better understand global patient experiences.

Further education on these topics is needed among clinicians who, as a result, may be better able to communicate with patients in a destigmatizing manner. A conceptual framework for opioid stigma has been developed for the context of cancer pain [75] and may be beneficial in designing interventions to address this barrier to pain management. The opioid stigma framework (OSF) is useful for designing health system interventions which may be targeted to specific domains, including intersecting stigmas, manifestations of stigma, and the impacts of opioid stigma for patients [75]. Further research is warranted to design interventions which address the underlying mechanisms of opioid stigma using this framework.

In other stigmatized domains, such as mental health, tailored multilevel interventions to educate and communicate about stigma have been found to be beneficial in alleviating negative perceptions [115]. Studies which employ interventions to reduce the stigma related to substance use disorders may additionally be helpful in designing strategies for cancer populations [116]. Clinicians, pharmacists and other healthcare professionals in this area should be aware of the highly stigmatized nature of opioid use in this setting and attempt to communicate with patients in ways that destigmatize effective pain management.

In addition to interventions directly targeting the foundations and consequences of opioid stigma, the effective treatment of cancer-related pain requires involvement of a multidisciplinary team [117]. It has been shown that the provision of palliative care can help to reduce the stigma surrounding appropriate pain management for patients with cancer [118]. Continued efforts should be made to integrate palliative care specialists, psychological support, pharmacists, and other healthcare professionals into patient-centered oncology care. Furthermore, improving the availability of substance use disorder or addiction specialists may be particularly useful for providing effective pain management to patients with a history of substance use disorders [119].

There is a need for personalized pain management strategies among patients with cancer, for whom both their pain and ability to manage it are modulated by individual factors such as mood, beliefs, culture, and pain etiology. Guidelines have been developed which encourage open conversations about patient goals and concerns regarding pain management which can facilitate personalized treatment decisions with the help of a multidisciplinary team [22]. However, these guidelines require targeted interventions to aid their successful implementation in routine cancer care. The integration of palliative, supportive, and oncology care may allow for cancer-related pain to be better assessed and addressed, as well as facilitate improved communication between patients and their team of healthcare professionals.

Additionally, the use of multimodal pain management strategies may aid in addressing pain undertreatment and the outcomes resulting from opioid stigma. The use of atypical opioid analgesics [120] or further research into non-opioid analgesia may be useful for improving pain management in these settings [121]. Furthermore, there is a growing body evidence for non-pharmacological management of cancer-related pain, including a variety of psychosocial interventions for patients with cancer [122,123], which provide additional evidence for the importance of multidisciplinary involvement in cancer management.

## 9. Conclusions

Opioids are an essential therapeutic option for symptom and pain management among patients with advanced cancer. However, the opioid crisis has led to highly publicized and widespread stigmatization of opioid use, which affects both patients and providers. While responses to the opioid crisis were not intended to affect the cancer population, stigma has transcended the intended scope of guidelines and become a significant barrier to pain management for patients with advanced cancer. Stigmatization manifests among healthcare professionals who provide access to opioids, and can result in disparities in access to resources among minorities or groups with increased stigma.

In spite of healthcare professionals being the gatekeepers of opioid analgesics, the most significant barriers to cancer pain management relate to the self-stigma experienced by patients. Patients with advanced cancer have fears related to addiction and diminishing returns, shame regarding their need for opioids, and difficulties communicating with providers about these topics. Self-stigma can act as a barrier to pain reporting, which makes physicians’ pain assessment impossible. This stigma may also act as a barrier to prescription adherence, which means that, even in the case of perfect prescribing practices, patients may not experience freedom from their pain.

Healthcare professionals should therefore be prepared to initiate and maintain conversations with patients such that stigma is reduced, not amplified or ignored. Specific and standardized risk assessment tools should take into account opioid stigma in order address imbalanced pharmacovigilance. Furthermore, accurate risk assessment for the advanced cancer population cannot be accomplished without a better understanding of the real-world risks of opioid use, which would allow for a well-informed benefit-to-harm framework for decision-making. More research is needed, not only to understand the experience of opioid stigma by these patients, but to provide complete information to patients, which demonstrates that they do not need to be afraid, shameful, and living with uncontrolled pain.
